# 1,3-Bis(4-methoxy­phen­yl)imidazolidium chloride monohydrate

**DOI:** 10.1107/S1600536808033965

**Published:** 2008-10-22

**Authors:** Yu Wan, Haiqiang Xin, Xiumei Chen, Huahong Xu, Hui Wu

**Affiliations:** a School of Chemistry and Chemical Engineering, Xuzhou Normal University, Xuzhou 221116, People’s Republic of China

## Abstract

The asymmetric unit of the title compound, C_17_H_17_N_2_O_2_^+^·Cl^−^·H_2_O, contains one-half of the cation, one-half of a water mol­ecule and a chloride anion. The complete cation is generated by crystallographic two-fold symmetry, with one C atom lying on the rotation axis.  The O and Cl atoms have site symmetry 2. The imidazolidium ring is oriented at a dihedral angle of 4.15 (3)° with respect to the 4-methoxy­phenyl ring and an intramolecular C—H⋯O interaction occurs. In the crystal structure, inter­molecular O—H⋯Cl and C—H⋯Cl hydrogen bonds link the mol­ecules. There is a π–π contact between the imidazolidium and 4-methoxy­phenyl rings [centroid-to-centroid distance = 3.625(3 Å]. There is also a C—H⋯π contact between the methyl group and the 4-methoxy­phenyl ring.

## Related literature

For general background, see: Lin & Vasam (2005 ▶). For bond-length data, see: Allen *et al.* (1987 ▶).
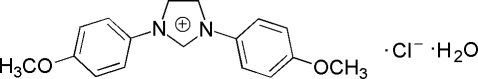


## Experimental

### 

#### Crystal data

C_17_H_17_N_2_O_2_^+^·Cl^−^·H_2_O
*M* *_r_* = 334.79
Monoclinic, 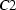

*a* = 15.6706 (19) Å
*b* = 9.4198 (9) Å
*c* = 5.4026 (4) Å
β = 90.156 (1)°
*V* = 797.50 (14) Å^3^
*Z* = 2
Mo *K*α radiation
μ = 0.26 mm^−1^
*T* = 298 (2) K
0.20 × 0.11 × 0.09 mm

#### Data collection

Bruker SMART CCD area-detector diffractometer
Absorption correction: multi-scan (*SADABS*; Sheldrick, 1996 ▶) *T*_min_ = 0.951, *T*_max_ = 0.977
2026 measured reflections
749 independent reflections
688 reflections with *I* > 2σ(*I*)
*R*_int_ = 0.021

#### Refinement

*R*[*F* ^2^ > 2σ(*F* ^2^)] = 0.029
*wR*(*F* ^2^) = 0.076
*S* = 1.01
749 reflections
107 parameters
1 restraint
H-atom parameters constrained
Δρ_max_ = 0.11 e Å^−3^
Δρ_min_ = −0.22 e Å^−3^

### 

Data collection: *SMART* (Bruker, 1998 ▶); cell refinement: *SAINT* (Bruker, 1999 ▶); data reduction: *SAINT*; program(s) used to solve structure: *SHELXS97* (Sheldrick, 2008 ▶); program(s) used to refine structure: *SHELXL97* (Sheldrick, 2008 ▶); molecular graphics: *ORTEP-3 for Windows* (Farrugia, 1997 ▶) and *PLATON* (Spek, 2003 ▶); software used to prepare material for publication: *SHELXTL* (Sheldrick, 2008 ▶) and *PLATON*.

## Supplementary Material

Crystal structure: contains datablocks global, I. DOI: 10.1107/S1600536808033965/hk2542sup1.cif

Structure factors: contains datablocks I. DOI: 10.1107/S1600536808033965/hk2542Isup2.hkl

Additional supplementary materials:  crystallographic information; 3D view; checkCIF report

## Figures and Tables

**Table 1:** Hydrogen-bond geometry (Å, °) *Cg*2 is the centroid of the C3–C8 ring.

*D*—H⋯*A*	*D*—H	H⋯*A*	*D*⋯*A*	*D*—H⋯*A*
O2—H2⋯Cl1	0.85	2.29	3.133 (3)	173
C1—H1⋯O2	0.93	2.13	3.060 (3)	180
C2—H2*A*⋯Cl1^i^	0.93	2.69	3.474 (3)	142
C4—H4⋯O2	0.93	2.47	3.391 (3)	170
C9—H9*C*⋯*Cg*2^ii^	0.96	2.91	3.629 (3)	133

Symmetry codes: (i) 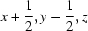; (ii) 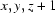.

**Fig. 1.**
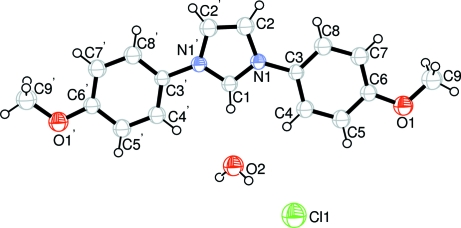
The molecular structure of the title molecule, with the atom-numbering scheme [symmetry code: (') -x, y, -z].

**Fig. 2.**
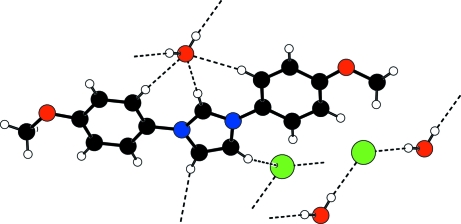
A partial packing diagram. Hydrogen bonds are shown as dashed lines.

## supplementary crystallographic information

### Comment

Imidazole and its derivatives such as imidazolium cation are important compounds playing important roles in medical, organic and material chemistry (Lin & Vasam, 2005). A broad application of imidazolium now is to synthesize ionic liquids. Recently, ionic liquids are attracting much attention as alternative reaction media for synthesis and catalysis. Its applications in many different areas including separation processes, catalyst, electrochemistry, electrolytes in solar cells and lubricants are widely recognized. Therefore, the need of ionic liquids with specific chemical and physical properties become stronger. We report herein the synthesis and crystal structure of the title compound.

The asymmetric unit of the title compound (Fig. 1) contains one half-molecule, one half-water molecule and a chloride atom. The bond lengths (Allen *et al.*,  1987) and angles are within normal ranges. Rings A (N1/N1'/C1/C2/C2') and B (C3–C8) are, of course, planar and the dihedral angle between them is A/B = 4.15 (3)° [symmetry code: (') -x, y, -z]. Intramolecular C—H···O and O—H···Cl hydrogen bonds (Table 1) link the molecules.

In the crystal structure, intramolecular C—H···O and O—H···Cl and intermolecular C—H···Cl hydrogen bonds (Table 1) link the molecules (Fig. 2), in which they may be effective in the stabilization of the structure. The π–π contact between the imidazolidium and 4-methoxyphenyl rings, Cg1···Cg2^i^ [symmetry code: (i) 1 - x, y, 1 - z, where Cg1 and Cg2 are the centroids of the rings A (N1/N1'/C1/C2/C2') and B (C3–C8), respectively] may further stabilize the structure, with centroid-centroid distance of 3.625 (3) Å. There also exist a C—H···π contact (Table 1) between the methyl group and the 4-methoxyphenyl ring.

### Experimental

The reaction of 4-methoxybenzenamine (2 mmol) with formaldehyde (aq. 37%, 1 mmol) and glyoxal (aq. 40%, 1 mmol) in ethanol (95%) at 273–278 K for 8 h afforded 1-(2,3-diethoxy-4-(4-methoxyphenyl)cyclopentyl)-4-methoxybenzene (yield; 89%). The title compound was obtained through the oxidization of 1-(2,3-diethoxy-4-(4-methoxyphenyl)cyclopentyl)-4-methoxybenzene by phosgene in DMF at 268–273 K (yield 95%).

### Refinement

H atoms were positioned geometrically, with O—H = 0.85 Å (for H_2_O) and C—H = 0.93 and 0.96 Å for aromatic and methyl H, respectively, and constrained to ride on their parent atoms with U_iso_(H) = xU_eq_(C,O), where x = 1.5 for methyl H and x = 1.2 for all other H atoms.

### Crystal data

**Table:**

C_17_H_17_N_2_O_2_^+^·Cl^−^·H_2_O	*F*(000) = 352
*M**_r_* = 334.79	*D*_x_ = 1.394 Mg m^−^^3^
Monoclinic, *C*2	Melting point = 492–494 K
Hall symbol: -C 2y	Mo *K*α radiation, λ = 0.71073 Å
*a* = 15.6706 (19) Å	Cell parameters from 1340 reflections
*b* = 9.4198 (9) Å	θ = 2.5–28.3°
*c* = 5.4026 (4) Å	µ = 0.26 mm^−^^1^
β = 90.156 (1)°	*T* = 298 K
*V* = 797.50 (14) Å^3^	Block, colourless
*Z* = 2	0.20 × 0.11 × 0.09 mm

### Data collection

**Table:**

Bruker SMART CCD area-detector diffractometer	749 independent reflections
Radiation source: fine-focus sealed tube	688 reflections with *I* > 2σ(*I*)
graphite	*R*_int_ = 0.021
φ and ω scans	θ_max_ = 25.0°, θ_min_ = 2.5°
Absorption correction: multi-scan (SADABS; Sheldrick, 1996)	*h* = −18→13
*T*_min_ = 0.951, *T*_max_ = 0.977	*k* = −9→11
2026 measured reflections	*l* = −6→6

### Refinement

**Table:**

Refinement on *F*^2^	Primary atom site location: structure-invariant direct methods
Least-squares matrix: full	Secondary atom site location: difference Fourier map
*R*[*F*^2^ > 2σ(*F*^2^)] = 0.029	Hydrogen site location: inferred from neighbouring sites
*wR*(*F*^2^) = 0.076	H-atom parameters constrained
*S* = 1.01	*w* = 1/[σ^2^(*F*_o_^2^) + (0.0503*P*)^2^ + 0.1521*P*] where *P* = (*F*_o_^2^ + 2*F*_c_^2^)/3
749 reflections	(Δ/σ)_max_ < 0.001
107 parameters	Δρ_max_ = 0.11 e Å^−^^3^
1 restraint	Δρ_min_ = −0.22 e Å^−^^3^

### Special details

**Table:**

Geometry. All e.s.d.'s (except the e.s.d. in the dihedral angle between two l.s. planes) are estimated using the full covariance matrix. The cell e.s.d.'s are taken into account individually in the estimation of e.s.d.'s in distances, angles and torsion angles; correlations between e.s.d.'s in cell parameters are only used when they are defined by crystal symmetry. An approximate (isotropic) treatment of cell e.s.d.'s is used for estimating e.s.d.'s involving l.s. planes.
Refinement. Refinement of *F*^2^ against ALL reflections. The weighted *R*-factor *wR* and goodness of fit *S* are based on *F*^2^, conventional *R*-factors *R* are based on *F*, with *F* set to zero for negative *F*^2^. The threshold expression of *F*^2^ > σ(*F*^2^) is used only for calculating *R*-factors(gt) *etc*. and is not relevant to the choice of reflections for refinement. *R*-factors based on *F*^2^ are statistically about twice as large as those based on *F*, and *R*- factors based on ALL data will be even larger.

### Fractional atomic coordinates and isotropic or equivalent isotropic displacement parameters (Å^2^)

**Table:**

	*x*	*y*	*z*	*U*_iso_*/*U*_eq_	
Cl1	0.5000	0.84927 (12)	0.5000	0.0800 (5)	
O1	0.24434 (13)	0.4740 (2)	0.9080 (3)	0.0519 (5)	
O2	0.5000	0.6808 (3)	0.0000	0.0567 (8)	
H2	0.5000	0.7335	0.1277	0.068*	
N1	0.45802 (12)	0.27396 (19)	0.1594 (3)	0.0334 (5)	
C1	0.5000	0.3560 (4)	0.0000	0.0350 (7)	
H1	0.5000	0.4547	0.0000	0.042*	
C2	0.47435 (17)	0.1342 (3)	0.0983 (5)	0.0444 (6)	
H2A	0.4534	0.0545	0.1795	0.053*	
C3	0.40340 (14)	0.3225 (3)	0.3567 (4)	0.0335 (5)	
C4	0.39574 (17)	0.4670 (3)	0.4036 (5)	0.0420 (6)	
H4	0.4262	0.5322	0.3094	0.050*	
C5	0.34270 (17)	0.5135 (3)	0.5903 (5)	0.0449 (6)	
H5	0.3378	0.6101	0.6225	0.054*	
C6	0.29642 (15)	0.4164 (3)	0.7309 (5)	0.0386 (6)	
C7	0.30558 (17)	0.2728 (3)	0.6859 (5)	0.0446 (6)	
H7	0.2758	0.2073	0.7812	0.054*	
C8	0.35934 (16)	0.2262 (3)	0.4979 (5)	0.0440 (6)	
H8	0.3654	0.1296	0.4678	0.053*	
C9	0.19340 (19)	0.3780 (4)	1.0497 (5)	0.0580 (8)	
H9A	0.1588	0.3219	0.9402	0.087*	
H9B	0.1573	0.4309	1.1594	0.087*	
H9C	0.2299	0.3168	1.1447	0.087*	

### Atomic displacement parameters (Å^2^)

**Table:**

	*U*^11^	*U*^22^	*U*^33^	*U*^12^	*U*^13^	*U*^23^
Cl1	0.1591 (13)	0.0423 (5)	0.0387 (5)	0.000	0.0069 (6)	0.000
O1	0.0545 (11)	0.0522 (12)	0.0491 (11)	0.0011 (9)	0.0158 (9)	0.0059 (9)
O2	0.092 (2)	0.0344 (15)	0.0434 (14)	0.000	0.0109 (14)	0.000
N1	0.0365 (11)	0.0278 (10)	0.0358 (10)	−0.0015 (8)	−0.0027 (8)	0.0024 (8)
C1	0.0400 (18)	0.0271 (15)	0.0379 (15)	0.000	0.0009 (14)	0.000
C2	0.0572 (16)	0.0300 (13)	0.0461 (13)	−0.0031 (11)	0.0030 (11)	0.0012 (11)
C3	0.0319 (12)	0.0362 (13)	0.0323 (10)	−0.0010 (10)	−0.0018 (9)	0.0024 (10)
C4	0.0473 (15)	0.0333 (14)	0.0455 (14)	−0.0024 (11)	0.0086 (11)	0.0080 (11)
C5	0.0510 (15)	0.0339 (13)	0.0499 (14)	0.0029 (12)	0.0085 (11)	0.0012 (12)
C6	0.0356 (14)	0.0442 (15)	0.0360 (12)	−0.0008 (11)	0.0001 (11)	0.0027 (11)
C7	0.0480 (15)	0.0421 (16)	0.0438 (14)	−0.0081 (12)	0.0045 (11)	0.0072 (12)
C8	0.0518 (16)	0.0319 (13)	0.0484 (15)	−0.0044 (12)	0.0012 (13)	0.0019 (12)
C9	0.0485 (16)	0.069 (2)	0.0566 (16)	−0.0036 (14)	0.0137 (13)	0.0109 (15)

### Geometric parameters (Å, °)

**Table:**

O1—C6	1.371 (3)	C4—C5	1.380 (4)
O1—C9	1.430 (3)	C4—H4	0.9300
O2—H2	0.8500	C5—C6	1.394 (4)
N1—C1	1.332 (3)	C5—H5	0.9300
N1—C2	1.382 (3)	C6—C7	1.382 (4)
N1—C3	1.443 (3)	C7—C8	1.392 (4)
C1—N1^i^	1.332 (3)	C7—H7	0.9300
C1—H1	0.9300	C8—H8	0.9300
C2—C2^i^	1.334 (5)	C9—H9A	0.9600
C2—H2A	0.9300	C9—H9B	0.9600
C3—C8	1.372 (3)	C9—H9C	0.9600
C3—C4	1.390 (4)		
			
C6—O1—C9	117.2 (2)	C4—C5—H5	119.8
C1—N1—C2	107.8 (2)	C6—C5—H5	119.8
C1—N1—C3	126.1 (2)	O1—C6—C7	124.9 (2)
C2—N1—C3	126.1 (2)	O1—C6—C5	115.6 (2)
N1^i^—C1—N1	109.1 (3)	C7—C6—C5	119.5 (2)
N1^i^—C1—H1	125.5	C6—C7—C8	120.0 (2)
N1—C1—H1	125.5	C6—C7—H7	120.0
C2^i^—C2—N1	107.63 (14)	C8—C7—H7	120.0
C2^i^—C2—H2A	126.2	C3—C8—C7	120.2 (2)
N1—C2—H2A	126.2	C3—C8—H8	119.9
C8—C3—C4	120.1 (2)	C7—C8—H8	119.9
C8—C3—N1	120.1 (2)	O1—C9—H9A	109.5
C4—C3—N1	119.8 (2)	O1—C9—H9B	109.5
C5—C4—C3	119.8 (2)	H9A—C9—H9B	109.5
C5—C4—H4	120.1	O1—C9—H9C	109.5
C3—C4—H4	120.1	H9A—C9—H9C	109.5
C4—C5—C6	120.4 (2)	H9B—C9—H9C	109.5
			
C2—N1—C1—N1^i^	0.12 (13)	C3—C4—C5—C6	−0.4 (4)
C3—N1—C1—N1^i^	−178.4 (2)	C9—O1—C6—C7	−2.5 (4)
C1—N1—C2—C2^i^	−0.3 (3)	C9—O1—C6—C5	177.7 (2)
C3—N1—C2—C2^i^	178.2 (2)	C4—C5—C6—O1	−178.7 (2)
C1—N1—C3—C8	175.65 (18)	C4—C5—C6—C7	1.5 (4)
C2—N1—C3—C8	−2.6 (3)	O1—C6—C7—C8	178.9 (2)
C1—N1—C3—C4	−4.5 (3)	C5—C6—C7—C8	−1.3 (4)
C2—N1—C3—C4	177.3 (2)	C4—C3—C8—C7	1.1 (3)
C8—C3—C4—C5	−0.9 (4)	N1—C3—C8—C7	−179.0 (2)
N1—C3—C4—C5	179.2 (2)	C6—C7—C8—C3	0.0 (4)

Symmetry codes: (i) −*x*+1, *y*, −*z*.

### Hydrogen-bond geometry (Å, °)

**Table:**

*D*—H···*A*	*D*—H	H···*A*	*D*···*A*	*D*—H···*A*
O2—H2···Cl1	0.85	2.29	3.133 (3)	173
C1—H1···O2	0.93	2.13	3.060 (3)	180
C2—H2A···Cl1^ii^	0.93	2.69	3.474 (3)	142
C4—H4···O2	0.93	2.47	3.391 (3)	170
C9—H9C···Cg2^iii^	0.96	2.91	3.629 (3)	133

Symmetry codes: (ii) *x*+1/2, *y*−1/2, *z*; (iii) *x*, *y*, *z*+1.
